# Mapping Young Adults’ Concerns and Attitudes toward Food-Related Sustainability Issues in Israel: Implications for Food Policy

**DOI:** 10.3390/nu12103190

**Published:** 2020-10-19

**Authors:** Sigal Tepper, Vered Kaufman-Shriqui, Danit Rivka Shahar

**Affiliations:** 1 Department of Nutritional Sciences, Tel Hai Academic College, Rd 9977, Upper Galilee 1220800, Israel; 2 Department of Nutrition Sciences, School of Health Sciences, Ariel University, Ariel 4076405, Israel; veredks@ariel.ac.il; 3 Department of Public Health, Ben-Gurion University of the Negev, Beer Sheva 8410501, Israel; dshahar@bgu.ac.il

**Keywords:** food policy, health policy, nutrition, obesity, sustainability

## Abstract

Identifying the concerns about and attitudes toward adopting a healthy, sustainable diet may facilitate the development of effective implementation policies targeted at changing an individual’s dietary choices toward reducing the environmental burden of food systems. This cross-sectional online study was conducted in Israel among 348 adults aged 20–45 who responded to an advertisement posted on several social media platforms. Respondents received a link for the survey after signing informed consent forms. The questionnaire included three sections: concerns regarding food-related sustainability issues, willingness to act (“self”), and expectation that leaders would act upon these issues (“leaders”). Responses were recorded on a 1–4 Likert scale. Health-related issues—healthy food and drink, food prices, food safety, and the quality of health services—were scored the highest, both in the “self” and “leaders” sections. In all items, the expectation that leaders would act was higher than the willingness to act (composite mean ± SD: 3.04 ± 3.11 vs. 2.51 ± 2.47, respectively, *p* < 0.001). There were significant differences among dietary patterns in all three components. Mapping young adults’ concerns about and attitudes toward food-related sustainability issues allows for the identification of leverages that can be further used as focus issues in messages and interventions such as communication, food labeling, and economic incentives.

## 1. Introduction

Unhealthy diets are an underlying cause of undernutrition, overweight, and obesity, contributing to non-communicable diseases and consequential mortality. These effects are interrelated with climate change [1,2]. For example, the obesity epidemic impacts climate change by the elevated energy consumption and consumption of high-energy foods; those in turn have been shown to be a major contributor to Greenhouse Gas (GHG) emissions [3]. Climate change impacts obesity by food supply changes, motorized transportation and physical inactivity due to extremes of weather. Climate change impacts food availability, contributing to undernutrition [4]. Simultaneously, excess human-made environmental processes take place, including massive urbanization, deforestation, and industrialization. All of these contribute to climate change and thus endanger the sustainability of food systems [5]. Common underlying societal factors may interact as barriers to or become opportunities for achieving changes [2].

The environmental impact of food accounts for 20–30% of greenhouse gas emissions, predominantly from animal-based food production [6]. This fact led the Food and Agriculture Organization of the United Nations (FAO) to advocate for the integration of healthy and sustainable food consumption advice into food-based national dietary guidelines [7]. Indeed, an increasing number of countries have already addressed this recommendation [8,9]. Recently, the EAT-Lancet Commission on Healthy Diets from Sustainable Food Systems published a reference diet that integrates both healthy and sustainable nutrition [5]. Numerous countries have examined how to adjust their local environments and policies to meet those recommendations, including Israel [10,11,12,13]. Implementing these recommendations within public health policy is still an issue [2,14]. Thus, it must be acknowledged that, like other social determinants that affect the development of obesity, personal food choices are influenced by upstream societal factors, which interact with individual factors to shape individual food consumption. Factors such as urbanization, changes in the types of employment, alterations to the food supply, policies, local legislation, trade agreements, and food taxes influence the availability and accessibility of the food from which individuals can eventually choose [8]. The European Commission’s communication “A Clean Planet for All” outlines the importance of changing consumer behavior to reach greenhouse gas emission neutrality by 2050 [15]. To date, different strategies have been suggested and implemented to achieve this goal. Some strategies, aimed at consumers, provide information to guide healthy food choices at the point of purchase. These include front-of-package labeling and mandated nutrition information [16,17,18]. Additional downstream interventions include economic incentives (e.g., taxation and subsidies) [19], interventions in schools and workplaces, and restrictions on advertisements [20].

The public’s compliance with these strategies depends, among other things, on attitudinal factors, e.g., norms, perceptions, beliefs, and values [21,22]. A better understanding of the perceptions and beliefs that drive the adoption of sustainable and healthy diets is essential for designing effective policies. Many studies focus on shifts in motivation and attitudes toward a healthy diet; however, there are few studies regarding motivation and attitudes for adopting more sustainable diets. These studies determined that beliefs about the impact of food on the climate correlate with the motivation to change dietary habits toward more sustainable ones [22,23]. Hence, when designing strategies, focusing on knowledge is insufficient for generating actions, and, as studies show, changing beliefs is more significant for achieving this goal [24]. Therefore, strategies should focus on both knowledge and changing beliefs.

Additional issues that play a role in forming beliefs and motivation for change include assessments of trust and credibility regarding scientists and other experts, perceptions of the role of government, and questions of individual versus collective responsibility [25]. These perceptions define how arguments regarding the impact of food choices on climate change are received and how these arguments interact with cultural and structural factors to shape the likelihood of behavior change. However, the literature on individuals’ perceptions about the role of government versus personal responsibility is limited.

In this paper, we explore Israeli young adults’ perceptions of food-related sustainability issues, which can be integrated into future policies. We asked what the main concerns of young adults with regard to food-related sustainability issues were, to what extent they were willing to take action, and what actions they expected from their leaders.

## 2. Methods

### 2.1. Study Design

The present cross-sectional survey was conducted online using a convenience sample. The survey simultaneously assessed the current participants’ diets, perceptions, and attitudes toward sustainable nutrition.

### 2.2. Study Population

The study population included all adult individuals who elected to complete the survey online and who provided informed consent following a phone interview. The purposes of the phone interview were the preliminary screening of respondents according to pre-defined subpopulations, and giving specific instruction on how to fill in the questionnaires.

### 2.3. Study Procedures

The survey was posted to public and personal social media pages and published in designated forums. Using personal connections and snowballing methodology, we approached subpopulations such as vegans and vegetarians; secular, orthodox Jews, and ultra-orthodox Jews (independent streams in Judaism differ by degrees of religiosity, self-identified); rural and urban participants; and potential respondents with various environmental orientations.

Although this was a convenience sample, we aimed for the study sample to be representative. Using data from the Central Bureau of Statistics in Israel, we included an equal ratio of men and women, approached cities’ and villages’ dwellers based on their distribution in Israel within the study’s age range, included secular and orthodox communities in the same ratio as in the general population, and had a range in terms of socioeconomic status [26]. Once achieving the representative sample for a sector, we excluded respondents from this sector at the phone interview.

Individuals could respond after a phone interview with the study coordinator. The study was approved by the ethics committee of Tel-Hai College, and all participants signed an informed consent form after the interview.

### 2.4. Data Acquisition and Survey Characteristics

Survey data were collected using the Qualtrics software version 11/2017 XM© web application (Provo, UT, USA, https://www.qualtrics.com). Participants received an email link to the questionnaire. The data were extracted into a CSV format and submitted for statistical analysis after quality assurance.

Sociodemographic and personal data included age, sex, employment status, marital status, academic education, religious identification, car ownership, smoking habits, weight status (self-reported), physical activity, and eating patterns. Employment status, marital status, academic education, and smoking were classified as binary variables and reported as percentages (employed —yes/no, married—yes/no, high education—yes/no, and smoking—yes/no). Physical activity included indoor and outdoor activity, both aerobic and anaerobic, reported as total hours per week. Eating patterns were self-reported as vegan, vegetarian, flexitarian, omnivorous, and highly animal-based diets, namely, ketogenic and paleo variations. The eating patterns were validated using food frequency questionnaires. Pescatarians were classified as flexitarians.

### 2.5. Food-Related Sustainability Questionnaire

The questionnaire included three parts: 1. food-related sustainability concerns (“To what extent are you personally concerned about the following issues?”), 2. attitudes concerning the importance of taking action on food-related health and sustainability issues (“To what extent it is important to you personally to acknowledge the following topics and to include them in your considerations, decisions and actions?”), and 3. Perceptions concerning the importance of actions taken by leaders and activists on food-related health and sustainability issues (“To what extent is it important to you that leaders around the world take action on the following topics and include them in their considerations, decisions and actions?”). The food-related sustainability concern items were based on Grunert et al. [27] and included 10 items. The items related to perceptions and attitudes surrounding actions taken to improve food-related health and sustainability items were modified from Van Loo et al. [9] and included 17 items. These 17 items were asked twice. First, the questions were phrased as “it is important that I act” and then as “it is important that leaders act”. Responses to the items were recorded on a 1–4 Likert scale ranging from “Taking action is not important to me at all” to “Taking action is very important to me”. The modifications of the questionnaires addressed the relatively low awareness of the word sustainability in Israel, and included a brief introduction on the definition of sustainability and an explanation of some questions. For example, the item “The environmental impact of food production” was explained in parentheses as “(Carbon dioxide emissions, air pollution, increased exploitation of lands and water)”. The item on food safety was also explained. The questionnaires were translated and modified toward Israeli concerns. Face validity was tested in order to check whether the items appeared to be measuring a construct that was meaningful to the respondents by three experts.

### 2.6. Food Frequency Questionnaire

To calculate the dietary intake of animal-based and plant-based protein, we used the food frequency questionnaire (FFQ) adapted for the Israeli population. The adaption and validation process for this questionnaire is described in detail elsewhere [28,29]. Of the total protein intake calculated from the FFQ, we computed the percent intakes of animal- and plant-based protein. The questionnaire was self-administered electronically, thus ensuring completeness of data, as submitting the questionnaire was not possible if any of the items were not answered.

### 2.7. Statistical Analysis

Data were downloaded from the Qualtrics software to Excel (Microsoft, Redmond, WA, USA) and analyzed with the SPSS v25 Statistical Analysis Software (IBM Corp., Armonk, NY, USA). The distributions of continuous variables were assessed for normality using the Kolmogorov–Smirnov test. Continuous variables are described as means and standard deviations. Nominal variables are presented as *n* (%). Associations between nominal variables were assessed using the chi-square test. For the comparison of mean scores, two-sample Wilcoxon rank tests or *t*-tests were used as appropriate. ANOVA was used to compare different dietary patterns. Cronbach’s alpha was used to assess the internal reliability and consistency of the multi-item scales. All analyses were two-sided and considered significant at *p* < 0.05.

## 3. Results

The survey included 348 participants. The characteristics of the study population are presented in Table 1. Participants were young adults (mean age ± SD: 32 ± 9.6), 51% (*n* = 179) were women, 70% declared normal weight status, and only 10% were current smokers. The living conditions and urbanization levels of the study participants were representative of the Israeli population. Higher rates of vegan/vegetarian or flexitarian were found among women and higher rates of animal-based nutrition among men (*p* < 0.0001).

The composite means of each of the questionnaires—concerns, self, and leaders—were associated with most of the demographic characteristics—gender, marital status, education, age, eating preferences, and drinking patterns (the frequency of drinking sugar-sweetened beverages and frequency of drinking low-calorie beverages). No associations were found between religious lifestyle and socioeconomic indices (persons per room and cars per person) (*p* < 0.05 for all coefficients).

### 3.1. Concerns Regarding Food-Related Sustainability Issues

The responses to the question “To what extent are you personally concerned about the following issues?” indicate that the participants were most concerned with the amount of wasted food, non-recyclable packages, and the environmental impact of the human use of lands and water for food production, and least concerned with the amount of energy used for transporting, cooking, and storing food. Overall, in terms of concerns, no gender differences were found, except for three items for which women were more concerned than men (Table 2). The composite mean ± SD of the total items was 2.43 ± 2.40. The ten items yielded a Cronbach’s alpha of 0.935, indicating a high degree of internal consistency of the ratings.

**Figure 1:**
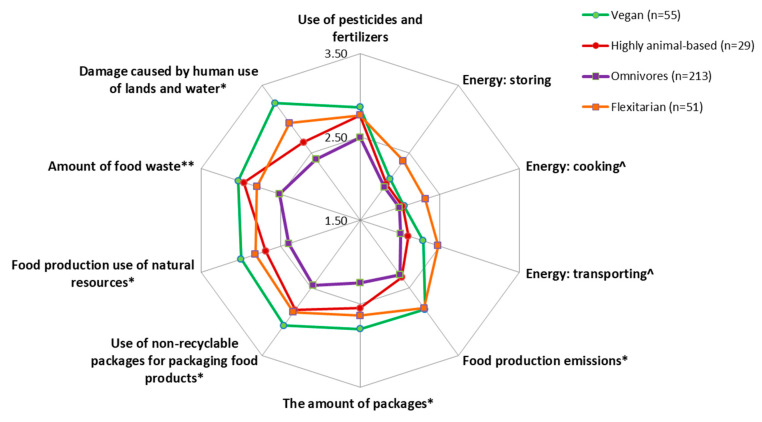
Food-related sustainability concerns by eating patterns. * Omnivores significantly differ from vegans and flexitarians, ** Omnivores significantly differ from vegans, ^ Omnivores significantly differ from flexitarians. All *p*-values < 0.05, corrected for Bonferroni multiple comparisons. Full results are in the Supplementary Table S1.

Next, we examined the association between eating patterns and food-related sustainability concerns. Figure 1 demonstrates that for most items, omnivorous consumers were less concerned with sustainability than vegans/vegetarians or flexitarians. The reported concern levels of consumers of animal-based diets (“paleo”) were similar to those held by vegans/vegetarians and flexitarians for most items.

### 3.2. Attitudes toward a Need to Act on a Personal or Public Leader Level

#### 3.2.1. Personal Level

Items related to “healthy food and beverages”, “food safety”, and “food prices” scored the highest. The participants were mostly motivated to take action on issues regarding health, food waste, and animal welfare, and least motivated on environmental issues. The composite mean ± SD of the total items was 2.51 ± 2.47. The 17 items yielded a Cronbach’s alpha of 0.931.

#### 3.2.2. Public Leaders

Items related to “food safety”, “quality of public health services”, “healthy food and beverages”, and “food prices” had the highest scores. Participants’ perceived importance was that leaders should act more on health and societal aspects than on environmental ones. The composite mean ± SD of the items was 3.04 ± 3.11. The 17 items yield a Cronbach’s alpha of 0.946.

#### 3.2.3. Comparison between “Self” and “Public Leaders”

**Figure 2:**
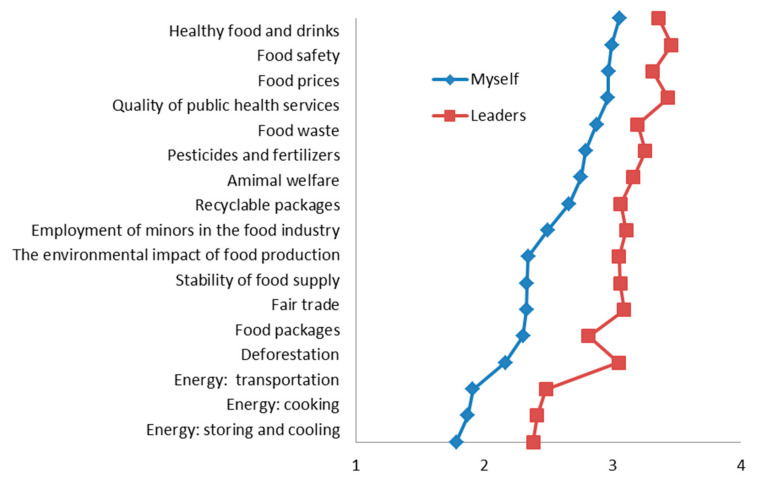
Perceptions of the importance of taking action on different aspects of food-related sustainability. Wilcoxon matched-pairs signed-rank test: all *p* < 0.0001 (for the difference between self and leaders).

The comparison between the respondents’ ranking of the personal and public leaders’ actions is presented in Figure 2. In general, higher scores for action were given to public leaders in comparison to the personal level. In both domains, the items ranked as having the highest importance to act on were “healthy food and beverages” and “food safety”. In both domains, the lowest scores were given to energy for storing, cooking, transportation, and deforestation.

### 3.3. Perceptions by Eating Patterns

**Figure 3:**
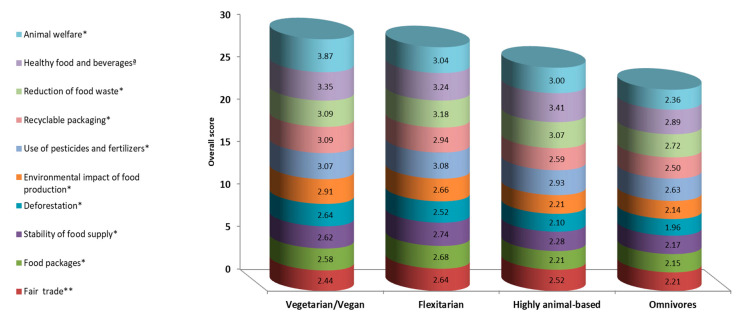
Willingness to act (perceptions of “it’s important that I act”) by eating pattern. Only significant items are shown. Items not included are employment of minors in the food industry, food prices, the quality of public health services, food safety, and energy for storing, cooking, and transporting. * Omnivores significantly differ from vegans and flexitarians. ** Omnivores significantly differ from flexitarians. ª Omnivores significantly differ from participants with all other patterns. All *p*-values < 0.05, Bonferroni-corrected for multiple comparisons.

The willingness to act differed according to the self-reported eating patterns. Omnivores reported significantly lower willingness to take action to promote healthier food and beverages than participants with all other dietary patterns (mean difference ± SE: −0.525 ± 0.163, *p* = 0.009). Highly animal-based participants reported significantly higher willingness to promote food safety (mean difference ± SE: 0.519 ± 0.185, *p* = 0.032). Generally, omnivores ranked most of the items lower than participants with other eating patterns. By contrast, flexitarians and vegetarians/vegans ranked most items higher than omnivores as well as the consumers of a highly animal-based diet (paleo) (Figure 3).

#### Participant Characterization by Dietary Patterns

Table 3 presents the characteristics of the participants by dietary patterns. The majority of the vegans/vegetarians and flexitarians were women, whereas most of the animal-based food eaters were men. The consumers of animal-based food were more physically active, and a higher percentage of them were employed and secular compared to omnivores and flexitarians. No differences in weight and smoking status were found. Food groups’ consumption across the dietary patterns demonstrated significant differences in the consumption of dairy, eggs, poultry, meat, fish, lentils, bread types, grains, vegetables, fruits, fats, and oils (*p* < 0.001) (Supplementary Figure S1, Table S1.)

## 4. Discussion

This paper explored young adults’ concerns about and perceptions of sustainable food-related topics. Our findings demonstrate that the participants perceive that it is highly important that policy makers act to promote sustainable food systems and, to a lesser extent, the importance of individual action. We have found that people were most concerned about food waste, the use of non-recyclable packages, and the environmental impact of the human use of lands and water for food production. They were the least concerned about the amount of energy used for transporting, storing, and cooking food products.

Previous research using similar items found somewhat different levels of concerns. Annunziata et al. [31] found that the use of pesticides in food production ranked first, followed by the amount of food waste and use of child labor in food production. Grunert et al. [27] found that the use of child labor in food production ranked first and deforestation second, reflecting the differences between countries. Overall, the total mean score of concerns was higher in studies conducted in Europe compared to ours. This may imply that people in Europe are more aware and concerned about food-related sustainability issues.

Our data illustrate an association between eating patterns, concerns, and attitudes toward food-related sustainability issues. The lower concerns of omnivores regarding food-related sustainability issues may reflect different attitudes to the association between dietary choices and climate change. Previous studies have shown that people have disbeliefs that dietary choices have an important influence on the environment [32] and that the impact of food is high compared to that of other non-food related behaviors (e.g., transportation) [33,34]. Despite ample evidence suggesting that a shift in consumers’ dietary patterns can improve health and the environment to a greater extent than other measures [5,35,36], there is still a gap between the scientific knowledge related to sustainable food policy strategies and individual knowledge and awareness. Numerous studies report that there is an underestimation of the impact of food choices on climate change in different countries [24,33,37,38]. Several studies have focused on possible change-directed actions. Mann et al. [32] found that established perceived health benefits or the perceived support of the local community increased people’s willingness to change food behavior. Lacroix and Gifford [39] also reported that even those who strongly opposed a reduction in meat consumption were willing to make a change of some sort toward healthier foods. These observations are in agreement with our findings. Those findings suggest that emphasizing potential individual health benefits and social values may be more influential than discussing environmental values.

While omnivores present a seemingly indifferent attitude toward food-related sustainability issues, flexitarians exhibited attitudes similar to those expressed by vegans/vegetarians on most items. Although, in practice, they have different eating patterns, our findings suggest they share common values. Flexitarian or semi-vegetarian diets are primarily vegetarian with the occasional inclusion of meat or fish. This diet has been clinically established as healthy and sustainable [40]. This indicates that although shifting toward healthier and more sustainable diets does mean a reduction in animal-based foods, especially meat [5,35], it does not necessarily mean shifting to a completely vegan or vegetarian diet. Policies aimed at triggering involvement in healthy and sustainable eating may have greater success if advocates shift toward the adaptation of flexitarian diets.

We have found a significant gap between the motivation to act and the perceived importance of policy makers acting to promote sustainable food systems. A multi-country survey conducted in Brazil, France, China, Russia, India, Germany, Italy, Japan, South Africa, Poland, the UK, and the USA found that participants with a greater awareness of the climate impact of meat and dairy consumption presented a markedly higher tendency to change their dietary choices. Knowledge of food-related climate change issues served as a precondition for both voluntary individual change and a positive response to government-led interventions encouraging dietary shifts [41].

Our findings highlight the importance of policy makers promoting healthy and sustainable food systems. Happer and Wellesley [25] analyzed the attitudes of focus groups toward changes in meat consumption in China, Brazil, the United Kingdom, and the United States. While there was an overall agreement that action should be taken and that governments have a responsibility to lead this action, the majority of participants raised concerns regarding politicians’ trustworthiness. Participants from the US emphasized the importance of ideological principles such as individual liberty and very limited government intervention in lifestyle choices. Nevertheless, there was a general reluctance identified among participants to accept their own active role in driving this change and a similar reluctance to accept responsibility for mitigating climate change. Notably, the researchers found an association between attitudes toward government actions and individual actions. Thus, a stronger sense of personal responsibility was associated with higher levels of trust in decision-makers [25].

### Suggested Strategies

The effective design of policies aimed at shifting dietary choices requires a better understanding of the motives underlying the adoption of sustainable and healthy diets. Our study, in agreement with other studies, [42,43], indicates that health issues matter more than environmental ones. Therefore, we suggest that health aspects should remain the main message when communicating and promoting healthy and sustainable diets. Furthermore, food safety and food prices were ranked high in the motivation to take action in our study and in agreement with others. Charlebois et al. [44] have identified that higher meat prices are a trigger factor in the reduction of consumption or avoidance of meat. Shang and Tonsor [45] have found that beef product recalls due to *E. coli* contamination significantly reduced consumer demand for these products. Price was also perceived as a barrier to shifting to healthier diets [23,46].

Investing efforts in public education to raise awareness of the consequences of dietary choices on the environment can increase public involvement and shift public preferences toward more sustainable and healthy choices [9,47,48]. While there is a general agreement on the use of education and nudging approaches to increase awareness and encourage behavioral change [49,50,51], the influence of economic steps such as regulations, taxation, and subsidies is debatable. Some of these strategies have been found to be more effective than soft approaches [52]. Subsidies appear to be more effective than taxes, although more information is needed [31].

Changing dietary patterns should be discussed in the context of local food preferences. Although Israel is a Mediterranean country, the majority of the Jewish population are first- to third-generation immigrants, and the dietary habits are still influenced by the immigrants’ countries of origin—mainly Europe. [28]. However, compared to that of the latter’s countries of origin, Israeli food is richer in fresh fruit, non-starchy vegetables, and olive oil [53]. The consumption of animal-based food is dominated by poultry [54]. Due to Jewish and Muslim religious restrictions, pork consumption is insignificant. Meat consumption has continuously increased since the beginning of the 21st century, ranking Israel fourth in the OECD for meat consumption [55]. These characteristics should be accounted for in future interventions.

The findings herein must be considered in the framework of the study limitations. First, the data are cross-sectional, and, as such, causality cannot be inferred. The associations between eating patterns and attitudes toward the sustainability of food patterns are correlational only. Second, the study population was a convenience sample, which limits generalizability and external validity. The major concern with a convenience sample is bias due to the under-representation or over-representation of particular groups within the sample. Nevertheless, the sampling method in our study assured the representation of the major subgroups in the population. Third, the relatively lower rates of smokers and of participants with overweight/obesity compared to those in the general population indicate that the study participants were healthier. Thus, the findings may be different in less healthy populations.

Despite its limitations, the present study used a validated FFQ to estimate individual intake and found interesting correlations between food patterns and perspectives on sustainable food systems. In addition, we used an internationally used questionnaire related to specific areas of concern and willingness to act to promote sustainable food systems, which allows us to draw local policy implications.

## 5. Conclusions

In conclusion, our study explored Israeli young adults’ concerns about and attitudes toward food-related sustainability issues and allows for the identification of leverage issues that can be further used to focus on messages and interventions such as communication, food labeling, and economic incentives.

## Figures and Tables

**Table 1:** Characteristics of the study population by gender.

		Women (*n* = 179)	Men (*n* = 169)	*p* ª	General Population ^1^
Age		32.54 ± 10.25	30.65 ± 8.73	0.068	
Married		80 (44.7%)	73 (43.2%)	0.799	45%
Secular		128 (71.5%)	119 (70.4%)	0.822	70%
Urbanization degree	City	98 (54.8%)	100 (59.2%)	0.71	60%
	Peripheral city	30 (16.8%)	27 (16%)		20%
	Village/community dwelling	51 (28.5%)	42 (24.8%)		
Persons per room		0.91 ± 0.34	0.9 ± 0.29	0.673	0.8
Employment status (working)		113 (63.1%)	91 (53.8%)	0.079	67%
**Health and Lifestyle**					
Weight status *	Underweight	8 (4.8%)	3 (1.8%)		
	Normal weight	117 (65.7%)	133 (79.1%)	0.041	
	Overweight	42 (23.6%)	29 (17.3%)		30.5%
	Obese	11 (6.2%)	3 (1.8%)		17%
Smoking (yes)		18 (10.1%)	18 (10.7%)	0.855	20%
Physical activity (hours per week)		3.1 ± 2.04	3.04 ± 2.37	0.835	
**Eating Patterns**					
Eating patterns	Vegetarian/vegan	40 (22.3%)	15 (9%)	<0.001	13%
	Flexitarian	35 (19.6%)	16 (9.5%)		23%
	Omnivores	93 (52%)	120 (71%)		
	Highly animal-based diet (paleo, ketogenic, etc.)	11 (6.1%)	18 (10.7%)		
Animal protein (%) ^∆^		54.27 ± 23.18	62.83 ± 21.39	0.001	

* Self-reported. Total of 346 (2 refused to answer). ^∆^ Animal protein vs. plant protein consumption was calculated from Food Frequency Questionnaire. ª *t*-test or chi-square *p*-value. ^1^ Age matched data [26,30].

**Table 2:** Food-related sustainability concerns ª by gender.

		Women (*n* = 179)		Men (*n* = 169)		Total		
Item#		Mean	Std	Mean	Std	Mean	Std	*p* *
1	The amount of food waste	2.76	0.86	2.59	0.1	2.68	0.93	0.095
2	Use of non-recyclable packages for packaging food products	2.71	0.89	2.57	0.93	2.64	0.91	0.170
3	Environmental damage caused by human use of lands and water for food production	2.70	0.1	2.56	1.0	2.63	0.96	0.155
4	The use of pesticides and fertilizers in food production	2.76	0.96	2.45	0.92	2.61	0.95	**0.002**
5	Using too much of the world’s natural resources for food production	2.65	0.89	2.50	0.94	2.58	0.92	0.130
6	Emissions caused by food production	2.59	0.96	2.33	0.95	2.46	0.96	**0.011**
7	The number of packages used in food products	2.47	0.94	2.36	0.91	2.42	0.93	0.264
8	The amount of energy used for transporting food products	2.23	0.89	2.02	0.90	2.13	0.89	**0.030**
9	The amount of energy used when storing food products	2.13	0.87	2.01	0.84	2.07	0.86	0.177
10	The amount of energy used when cooking food products	2.14	0.86	1.96	0.87	2.05	0.87	0.067

Score per item ranged from 1 to 4; overall, the total score for concerns ranged from X to Y. * *p* for differences between women and men. ª Total mean sorted in descending order. Bolded *p*-values indicate significant findings at *p* < 0.05.

**Table 3:** Characterization of participants by eating patterns.

	Vegetarian/Vegan (*n* = 55)	Flexitarian (*n* = 51)	Omnivore (*n* = 213)	Animal-Based Food (*n* = 29)	Total (*n* = 348)	*p* ª
Age	31.78 ± 8.99	34.6 ± 9.96	30.23 ± 9.23	34.97 ± 9.76	31.53 ± 9.49	0.004
Gender (women)	40 (73%)	35 (69%)	90 (42%)	11 (38%)	176 (51%)	<0.0001
Secular	52 (95%)	40 (78%)	126 (59%)	26 (90%)	244 (70%)	<0.0001
Persons per room	0.8 ± 0.28	0.91 ± 0.28	0.93 ± 0.32	0.96 ± 0.38	0.91 ± 0.32	0.037
Employment status (working)	34 (64%)	32 (63%)	109 (51%)	26 (90%)	201 (58%)	0.005
Education (academic)	12 (22%)	9 (18%)	52 (24%)	6 (21%)	79 (23%)	0.850
Weight status (normal)	45 (82%)	40 (78%)	146 (69%)	17 (59%)	248 (71%)	0.152
Smoking (yes)	5 (9%)	9 (18%)	19 (9%)	3 (10%)	36 (10%)	0.429
Physical activity (hours per week)	3.53 ± 2.67	2.46 ± 1.33	2.95 ± 2.2	4.06 ± 2.39	3.07 ± 2.22	0.024
Animal protein ^∆^ (%)	18.06 ± 19.16	50.88 ± 13.69	67.6 ± 10.58	79.59 ± 7.35	58.33 ± 22.78	<0.0001

Data presented as mean ± SD or number (percent). ^∆^ Animal protein vs. plant protein consumption calculated from FFQ. The source of animal protein for vegetarians is dairy products. ª ANOVA or chi-square *p*-value. SD: Standard Deviation.

## Supplementary Materials

The following are available online at https://www.mdpi.com/2072-6643/12/10/3190/s1, Figure S1: Food group consumption (FFQ) by dietary patterns, Table S1: Differences in consumption of selected food groups.
