# Supplementary material for: Mapping Young Adults’ Concerns and Attitudes toward Food-Related Sustainability Issues in Israel: Implications for Food Policy
Source: Nutrients. 2020 Oct 19;12(10):3190. doi: 10.3390/nu12103190 (PMC7603225; doi:10.3390/nu12103190)
Supplement: Supplementary file 1 [file nutrients-12-03190-s001.pdf]

Supplementary:

Figure S1: Food group consumption (FFQ) by dietary patterns:

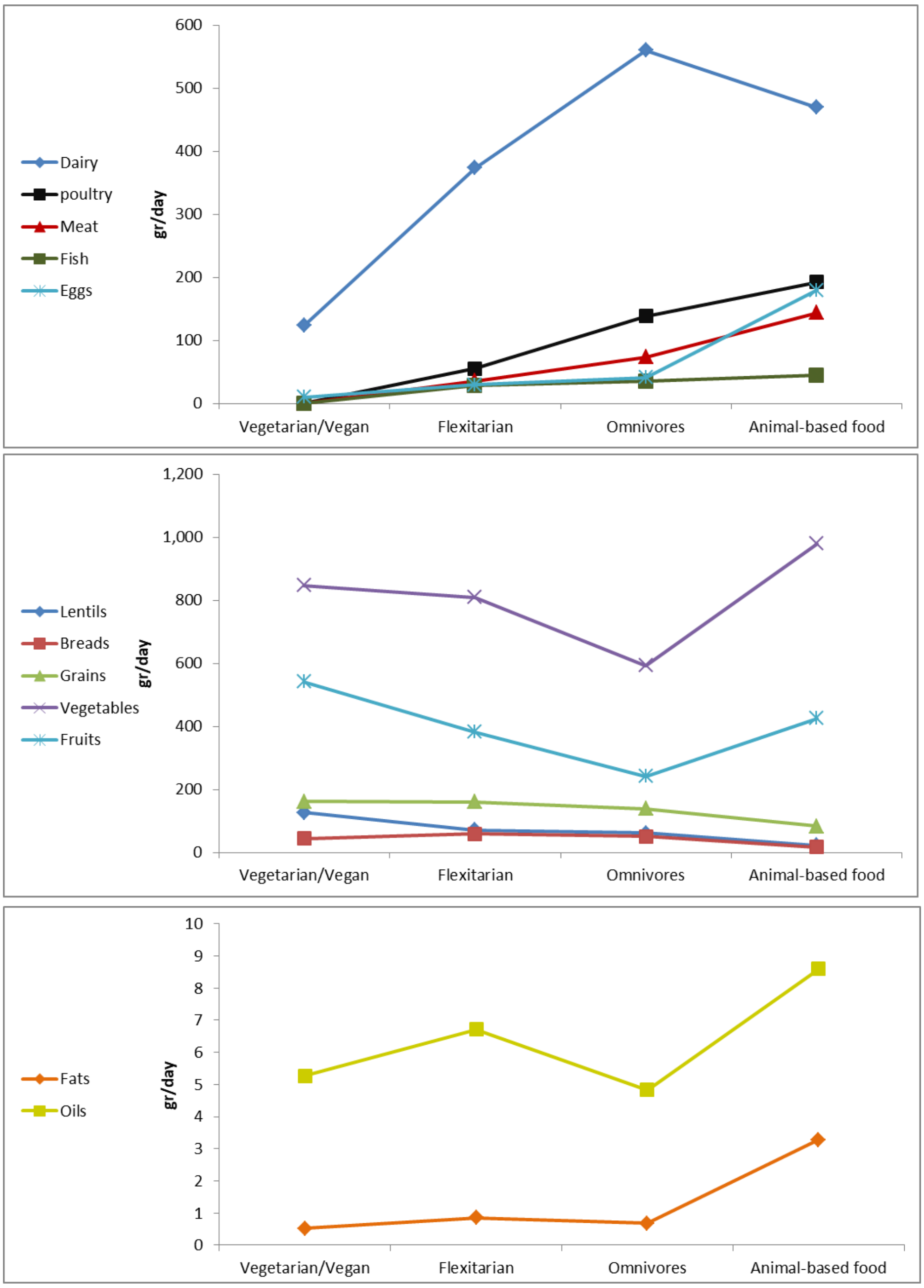

Table S1.: Differences in consumption of selected food groups:.

	Vegetarian/Vegan		Flexitarian		Omnivores		Animal-based food		
	**Mean**	**Std. Deviation**	**Mean**	**Std. Deviation**	**Mean**	**Std. Deviation**	**Mean**	**Std. Deviation**	**Sig***
**Dairy consumption**	123.5	254.8	373.5	336.8	560.3	542.8	469.7	1136.2	0.000
**Eggs consumption**	10.4	20.4	29.3	22.1	41.0	49.2	179.8	179.9	0.000
**poultry consumption**	0.0	0.0	55.2	36.6	138.4	111.2	192.2	143.5	0.000

**Meat consumption**	0.0	10.3	35.5	63.0	73.4	76.9	143.6	129.2	0.000
**Fish consumption**	2.2	5.6	28.2	43.3	35.3	58.8	44.5	49.9	0.000
**Lentils consumption**	126.4	107.6	71.0	53.3	61.9	117.0	22.5	29.4	0.000
**Breads consumption**	44.4	51.7	59.1	50.4	51.5	43.1	16.7	36.4	0.000
**Grains consumption**	162.5	126.5	160.6	134.5	139.6	99.0	83.7	78.7	0.009
**Vegetables consumption**	847.5	629.0	809.6	613.8	593.4	526.6	979.2	731.5	0.000
**Fruits consumption**	541.9	635.0	382.0	329.0	241.1	212.1	426.0	295.2	
**Alcohol consumption**	37.2	47.0	53.4	75.4	41.9	57.8	81.7	89.6	0.009
**Fats consumption**	0.5	1.2	0.9	1.2	0.7	1.3	3.3	5.0	0.000
**Oils consumption**	5.3	4.9	6.7	4.6	4.8	4.9	8.6	5.1	0.000

*Anova
